# Cancer stem cell-related gene expression as a potential biomarker of response for first-in-class imipridone ONC201 in solid tumors

**DOI:** 10.1371/journal.pone.0180541

**Published:** 2017-08-02

**Authors:** Varun V. Prabhu, Amriti R. Lulla, Neel S. Madhukar, Marie D. Ralff, Dan Zhao, Christina Leah B. Kline, A. Pieter J. Van den Heuvel, Avital Lev, Mathew J. Garnett, Ultan McDermott, Cyril H. Benes, Tracy T. Batchelor, Andrew S. Chi, Olivier Elemento, Joshua E. Allen, Wafik S. El-Deiry

**Affiliations:** 1 Oncoceutics, Inc., Philadelphia, Pennsylvania, United States of America; 2 Fox Chase Cancer Center, Philadelphia, Pennsylvania, United States of America; 3 Penn State College of Medicine, Hershey, Pennsylvania, United States of America; 4 Weill Cornell Medicine, New York, New York, United States of America; 5 Massachusetts General Hospital, Harvard Medical School, Boston, Massachusetts, United States of America; 6 Wellcome Trust Sanger Institute, Hinxton, United Kingdom; Università degli Studi della Campania "Luigi Vanvitelli", ITALY

## Abstract

Cancer stem cells (CSCs) correlate with recurrence, metastasis and poor survival in clinical studies. Encouraging results from clinical trials of CSC inhibitors have further validated CSCs as therapeutic targets. ONC201 is a first-in-class small molecule imipridone in Phase I/II clinical trials for advanced cancer. We have previously shown that ONC201 targets self-renewing, chemotherapy-resistant colorectal CSCs via Akt/ERK inhibition and DR5/TRAIL induction. In this study, we demonstrate that the anti-CSC effects of ONC201 involve early changes in stem cell-related gene expression prior to tumor cell death induction. A targeted network analysis of gene expression profiles in colorectal cancer cells revealed that ONC201 downregulates stem cell pathways such as Wnt signaling and modulates genes (*ID1*, *ID2*, *ID3* and *ALDH7A1*) known to regulate self-renewal in colorectal, prostate cancer and glioblastoma. ONC201-mediated changes in CSC-related gene expression were validated at the RNA and protein level for each tumor type. Accordingly, we observed inhibition of self-renewal and CSC markers in prostate cancer cell lines and patient-derived glioblastoma cells upon ONC201 treatment. Interestingly, ONC201-mediated CSC depletion does not occur in colorectal cancer cells with acquired resistance to ONC201. Finally, we observed that basal expression of CSC-related genes (*ID1*, *CD44*, *HES7* and *TCF3*) significantly correlate with ONC201 efficacy in >1000 cancer cell lines and combining the expression of multiple genes leads to a stronger overall prediction. These proof-of-concept studies provide a rationale for testing CSC expression at the RNA and protein level as a predictive and pharmacodynamic biomarker of ONC201 response in ongoing clinical studies.

## Introduction

Several clinical studies have demonstrated the relevance of cancer stem cells (CSCs) that clearly correlate with recurrence, metastasis and poor survival in solid tumors [1–3]. Recent objective responses observed in Phase I/II clinical trials of various CSC-targeted agents in a number of advanced refractory solid tumors have further established the importance of CSCs as a therapeutic target [4–6].

The first-in-class small molecule imipridone ONC201 is currently in Phase I/II clinical trials for advanced cancer [7]. The first-in-human Phase I study in advanced solid tumors demonstrated ONC201 to be safe, and exhibit predicted pharmacokinetics, sustained pharmacodynamics and tumor shrinkage [8]. The anti-CSC efficacy of ONC201 has been previously demonstrated *in vitro* and *in vivo* in colorectal cancer and acute myeloid leukemia (AML) [9, 10]. ONC201-mediated depletion of chemotherapy-resistant colorectal CSCs involves dual inactivation of Akt and ERK signaling that results in transcription factor Foxo3 activation that leads to DR5/TRAIL-dependent inhibition of self-renewal [9, 11]. In the current study, we evaluated whether the anti-CSC effects of ONC201 involve early changes in stem-cell related gene expression prior to tumor cell death. We examined if ONC201-mediated inhibition of CSCs extends to other solid tumors. Additionally, we tested whether CSC expression can serve as a potential biomarker of ONC201 response.

## Materials and methods

### Cell culture and reagents

HCT116 p53-/- cells were kind gifts from Dr. Bert Vogelstein of Johns Hopkins University. ONC201 resistant RKO cells were generated previously in our lab in 2012–2013 [12]. All other cell lines were obtained from the American Type Culture Collection and cultured as previously described [11, 12]. Cells were authenticated every month by growth and morphological observation. ONC201 was provided by Oncoceutics, Inc.

### Tumorsphere culture

Tumorspheres were cultured as described previously [9] under non-adherent growth conditions in Ultra Low attachment plates (Corning) using the MammoCult^™^ Human Medium (STEMCELL Technologies) as per the manufacturer’s protocol. Cells (1000–20,000 per well) were seeded medium containing DMSO or ONC201. Colonospheres of size > 60 μm were counted.

### Patient-derived glioblastoma cells

Four lines were derived using neurosphere culture from untreated (GBM8, GBM18) and recurrent (GBM67R and GBM152) glioblastomas. Cell viability assays were performed using indicated concentrations of ONC201 and IC50 values were calculated.

### Gene expression profiling and network analysis

Gene expression profiling of HCT116, RKO and ONC201-resistant RKO cells with DMSO or ONC201 treatment for indicated time points was performed in previous studies and data from these microarray studies are submitted to NCBI Gene Expression Omnibus [11, 12]. For network analysis of stem cell-related transcriptional changes induced by ONC201, the dataset was analyzed with the Ingenuity Pathway Analysis software.

### Quantitative RT-PCR (qRT-PCR)

Total RNA was isolated using the Quick-RNA^™^ MiniPrep kit (Zymo Research, Irvine, CA). 5μg of total RNA from each sample was subjected to cDNA synthesis using SuperScript^®^ III Reverse Transcriptase kit (Life technologies, Grand Island, NY). The relative expression of the reported stem-cell markers was determined using real-time PCR performed on Applied Biosystems 7900HT Fast Real-Time PCR system. Each cDNA sample was amplified using Power SYBR Green (Applied Biosystems, CA). Briefly, the reaction conditions consisted of 0.4 μL of cDNA and 0.2 μM primers in a final volume of 10 μL of qPCR mix. Each cycle consisted of denaturation of 95°C for 15 s, annealing at 60°C for 15 s and extension at 72°C for 1 min. Each cycle was followed by dissociation curves for every sample. The primers for the markers are listed in S1 Table. GAPDH was used as an endogenous control to normalize each sample. At least two different independent experiments were performed for each result with triplicates per experiment.

### Western blot

Western blotting was performed as described previously [9, 11, 12]. The following antibodies were used: CD44 (Cell Signaling), ALDH (BD), ID1 (Santa Cruz), ID2 (Santa Cruz), ID3 (Santa Cruz), CD133 (Santa Cruz Biotechnology), WNT16 (BD) and Ran (BD). Horseradish peroxidase labeled secondary antibodies were from Pierce.

### Analysis of gene expression data from genomic of drug sensitivity in cancer (GDSC) cell line screening

Cell viability assays were performed with GDSC cell lines (1000 human cancer cell lines) at 72 hours post-ONC201 treatment to generate dose responses curves at concentrations from 78 nM up to 20 μM as described previously [7]. Gene expression data was downloaded from the COSMIC Cell Lines Project using an Affymetrix Human Genome U219 Array platform. GDSC cell lines were separated in low and high expression groups based on a Z-score cutoff of -1 and 1 respectively. Data were analyzed to generate IC50. A Kolmogorov—Smirnov test (using the ks.test method in the R statistical programming language) was used to test statistical significance with the accompanying D statistic used to measure the degree of separation between the two groups.

### Other statistical analysis

Data are presented as the mean ± standard deviation or standard error of mean from at least three replicates. The Student’s two-tailed t-test in Excel (Microsoft) was used for pairwise analysis. Statistically significant changes (*) are indicated in the figures with p-values.

## Results

### ONC201 modulates stem cell-related gene expression

**Fig 1:**
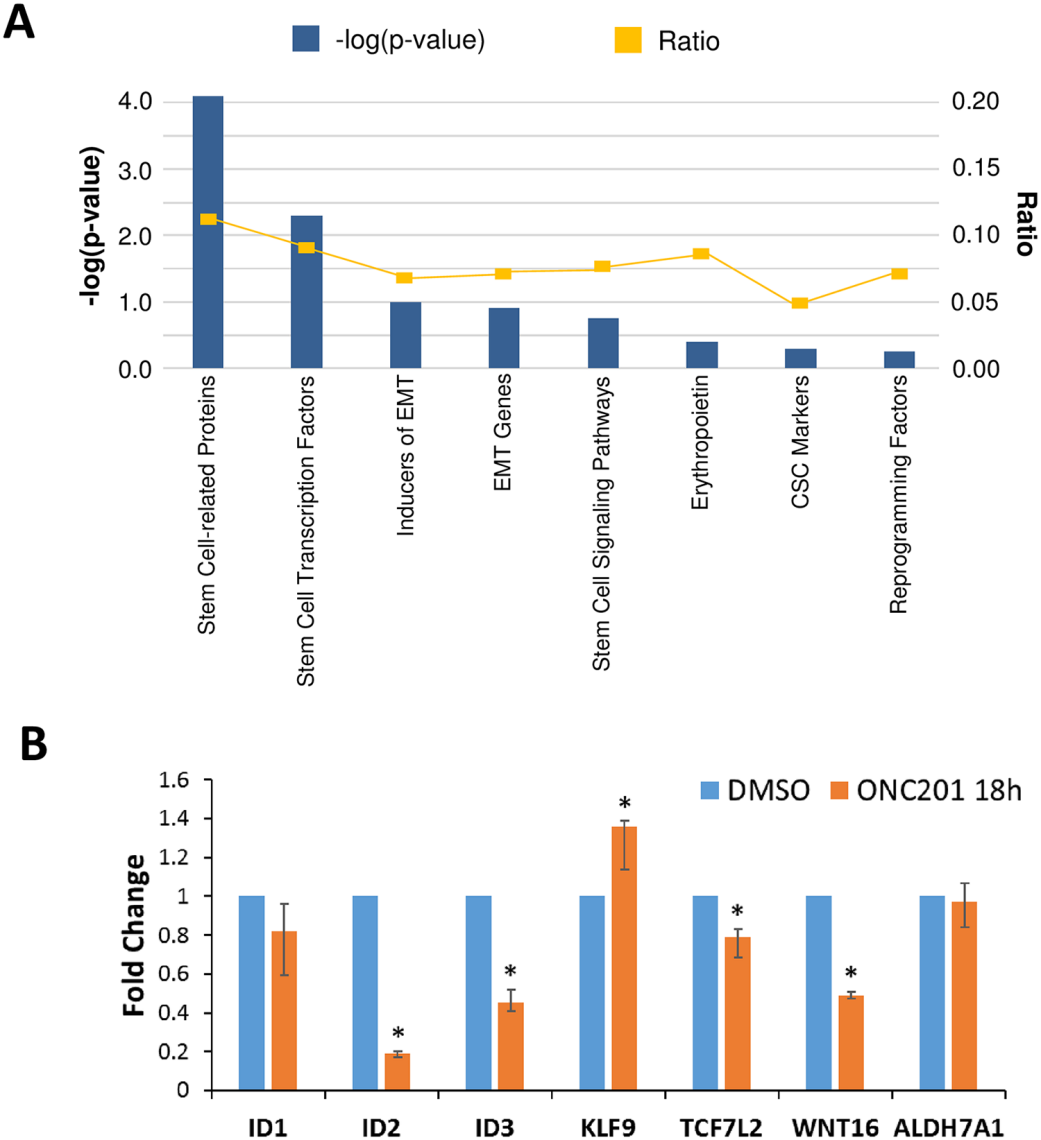
ONC201 modulates stem cell-related gene expression. (A) Summary of targeted network analysis of stem cell-related changes in ONC201-treated (10 μM) HCT116 p53-null cells by Ingenuity Pathway Analysis. The—log(p-value) is indicated for each group of genes. Ratio indicates the relative number of genes that were significantly changed upon ONC201 treatment compared to total number of genes in the group. (B) qRT-PCR for indicated stem cell-related genes in DMSO/ONC201-treated (5 μM, 18 h/48 h, n = 3) HCT116 p53-null cells. * indicates p < 0.02 relative to DMSO.

A targeted network analysis of gene expression profiles of HCT116 p53-null human colon cancer cells treated with ONC201 (18 h and 48 h) revealed that several stem cell-related genes, transcription factors and signaling pathways are significantly modulated by the compound (Fig 1A and S2–S4 Tables). Specifically, mRNA levels of *ID1* (colon/glioblastoma CSC-regulation [13], 2.5-fold), *ID2* (glioma stem cell regulation [13], 3.2-fold), *ID3* (colon/glioma CSC-regulation [13], 2.9-fold), *ALDH7A1* (prostate CSC marker/metastasis [14], 2-fold) were significantly downregulated and *KLF9* (glioblastoma stem cell inhibitor [15], 1.5-fold) was significantly upregulated in HCT116 p53-null cells upon 48 hour ONC201 treatment (Table 1), indicative of potential anti-CSC effects in these solid tumors. Also, mRNA levels of Wnt pathway-related genes such as ligand *WNT16* (hematopoietic stem cell [16]/prostate cancer resistance-related [17], 13.5-fold), receptors *FZD2* (regulator of epithelial-mesenchymal transition (EMT)/colon cancer metastasis [18], 2.98-fold), *FZD4* (glioma stemness [19], 3.9-fold) and transcription factor *TCF7L2* (stem cell differentiation [20], 3.55-fold) were significantly downregulated (Table 1). Genes involved in Wnt signaling, Hedgehog signaling and stem cell pluripotency were significantly modulated as early as 18 h upon ONC201 treatment (Table 2 and S4 Table). Modulation of stem cell-related transcription was further confirmed in RKO colorectal cancer cells upon ONC201-treament (48 h) (Table 2 and S5 Table). Validation with qRT-PCR indicated that *ID2*, *ID3*, *TCF7L2*, *WNT16* mRNA levels were significantly downregulated while *KLF9* mRNA was significantly upregulated in response to ONC201 treatment (18 h) in HCT116 p53-null cells (Fig 1B). Clearly, ONC201 specifically impacts stem cell-related transcription at time points (18 and 48 h) that precede cell death, which occur beyond 48 h in solid tumor cells [11]. These early effects on stem-cell related transcription are followed by inhibition of CSC markers and self-renewal by ONC201 at 48–72 h [9].

**Table 1:** ONC201-mediated CSC- and Wnt-pathway-related changes in gene expression.

**Gene**	**Fold change**	**mRNA Level**	**P value**	**CSC function**
*ALDH7A1*	2.002995	down	3.16E-03	Prostate CSC marker
*ID1*	2.518517	down	5.85E-04	Colorectal/glioblastoma CSC-related protein
*ID2*	3.2357852	down	8.49E-05	Glioma stem cell-related protein
*ID3*	2.884441	down	1.05E-03	Colorectal/glioma CSC-related protein
*KLF9*	1.542067	up	4.88E-03	Glioblastoma stem cell-related protein
*WNT16*	13.495952	down	1.98E-03	Prostate Cancer Resistance, HSC regulation
**Gene**	**Fold change**	**mRNA Level**	**P value**	**Wnt pathway function**
*WNT16*	13.495952	down	1.98E-03	Ligand
*FZD2*	2.989978	down	8.61E-04	Receptor
*FZD4*	3.9322994	down	1.38E-03	Receptor
*TCF7L2*	3.5501168	down	5.11E-03	Transcription factor

CSC- and Wnt pathway-related drug-induced changes identified with Ingenuity Pathway Analysis for gene expression profiles of HCT116 p53-null cells treated with ONC201 (10 μM) for 48 h. Fold change relative to DMSO treated cells.

**Table 2:** ONC201-mediated stem cell-related changes in gene expression.

RKO cells treated with ONC201 48h	**Molecules**	**-log(p-value)**	**Ratio**	**Ingenuity Canonical Pathways**
	*RAF1*, *JAK1*, *MAPK1*, *RRAS*, *PIK3R1*, *HRAS*, *SMAD5*, *TCF3*, *PIK3R4*, *ID1*, *FZD4*, *PIK3C3*, *MAP2K1*	3.72E-01	1.31E-01	Mouse Embryonic Stem Cell Pluripotency
	*ARRB2*, *PRKAR1B*, *CDK1*, *CCNB1*	2.94E-01	1.21E-01	Sonic Hedgehog Signaling
	*APH1B*, *LFNG*, *HES7*, *DTX2*, *HEY1*	2.93E-01	1.22E-01	Notch Signaling
HCT116 cells treated with ONC201 18h and 48h	**Molecules**	**-log(p-value)**	**Ratio**	**Ingenuity Canonical Pathways**
	*ID1*, *ID2*, *FZD4*, *BMPR2*, *ID3*, *FZD2*, *TCF7L2*	7.2E-01	7.07E-02	Mouse Embryonic Stem Cell Pluripotency
	*CDK1*, *CCNB1*	3.72E-01	6.06E-02	Sonic Hedgehog Signaling
	*FZD4*, *HDAC1*, *BMPR2*, *WNT16*, *KREMEN2*, *PIN1*, *DKK1*, *FZD2*, *TCF7L2*	3.31E-01	5.17E-02	Wnt/β-catenin Signaling

Stem cell-related pathways identified with Ingenuity pathway analysis for gene expression profiles of tumor cells treated with ONC201 (5 μM).

### ONC201 targets CSCs in prostate and glioblastoma tumors

**Fig 2:**
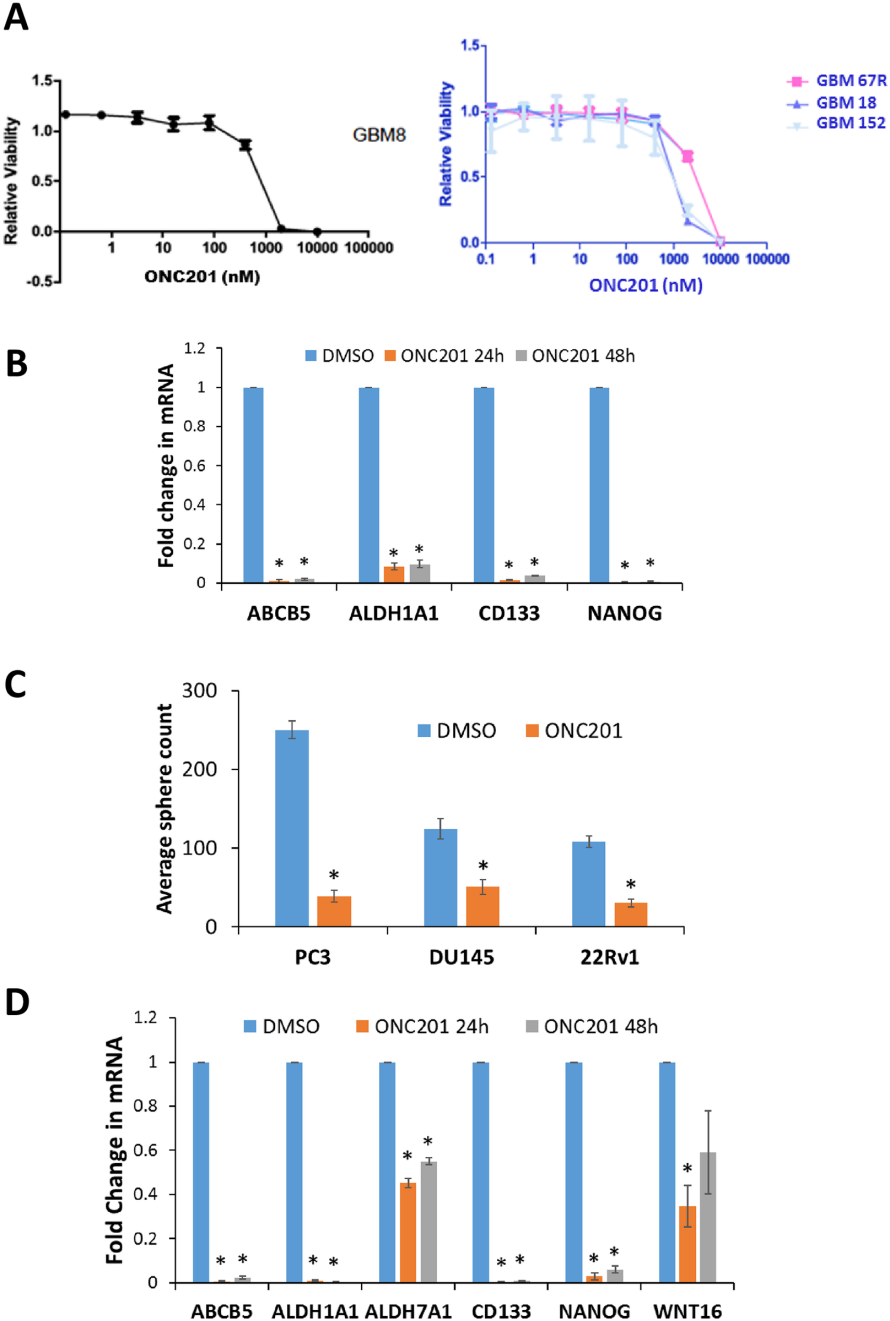
ONC201 targets CSCs in prostate and glioblastoma tumors. (A) Effect of indicated concentrations of ONC201 (72 h) on viability of newly diagnosed (GBM8, GBM18) and recurrent (GBM67R, GBM 152) glioblastoma cells in 3D neurosphere culture. (B) qRT-PCR for indicated stem cell-related genes in DMSO/ONC201-treated (5 μM, 24 h/48 h, n = 3) SNB19 cells. * indicates p < 0.0002 relative to DMSO. (C) Effect of DMSO/ONC201 (5 μM, 72 h, n = 3) on tumor sphere formation of indicated prostate cancer cell lines. * indicates p < 0.025 relative to DMSO. (D) qRT-PCR for indicated stem cell-related genes in DMSO/ONC201-treated (5 μM, 24 h/48 h, n = 3) DU145 cells. * indicates p < 0.04 relative to DMSO.

Based on the relevance of the CSC-related genes modulated by ONC201 in prostate cancer and glioblastoma, we tested the effects of ONC201 on CSC-related gene expression and self-renewal in these tumor types. ONC201 was tested in CSC-enriched 3-dimensional neurosphere culture models of primary glioblastoma samples, including newly diagnosed (GBM8, GBM18) and recurrent (GBM67R and GBM152) samples. ONC201 potently inhibited *in vitro* cell viability of all 4 lines, with IC50 values of 433 nM (GBM18), 1.09 μM (GBM8), 3.97 μM (GBM67R) and 688 nM (GBM152) (Fig 2A). We have previously demonstrated that ONC201 downregulated CSC markers *CD133*, *ALDH1A1* and *CD44* in colorectal cancer cells *in vitro* and *in vivo* [9]. Consistent with these findings, ONC201 significantly downregulated CSC-related genes *ABCB5*, ALDH1A1, *CD133* and *NANOG* in SNB19, T98G and U251 glioblastoma cells (Fig 2B and S1 Fig). Western blotting showed that *CD133*, *ALDH1*, *NANOG*, *ID1* and *ID3* were downregulated in U251 and T98G glioblastoma cells upon ONC201 treatment at 72 h (S1 Fig). *ID1* protein is upregulated at 24 h (S1 Fig), however, mRNA levels decrease at 48 h (Table 1) and protein levels decrease by 72 h post ONC201 treatment (S1 Fig). ONC201 significantly reduced tumorsphere formation of 22Rv1, DU145 and PC3 human prostate cancer cells (Fig 2C). ONC201 significantly downregulated CSC-related genes *ABCB5*, *ALDH1A1*, *ALDH7A1*, *WNT16*, *CD133* and *NANOG* in DU145 prostate cancer cells (Fig 2D). Western blotting revealed that *WNT16* was downregulated in LNCaP and 22Rv1 while CSC marker *CD44* was downregulated in 22Rv1 cells upon ONC201 treatment at 72 h (S1 Fig). Thus, changes in stem cell-related transcription and anti-CSC effects of ONC201 observed in colorectal cancer extend to prostate cancer and glioblastoma.

### Inhibition of CSCs does not occur in tumor cells with acquired resistance to ONC201

**Fig 3:**
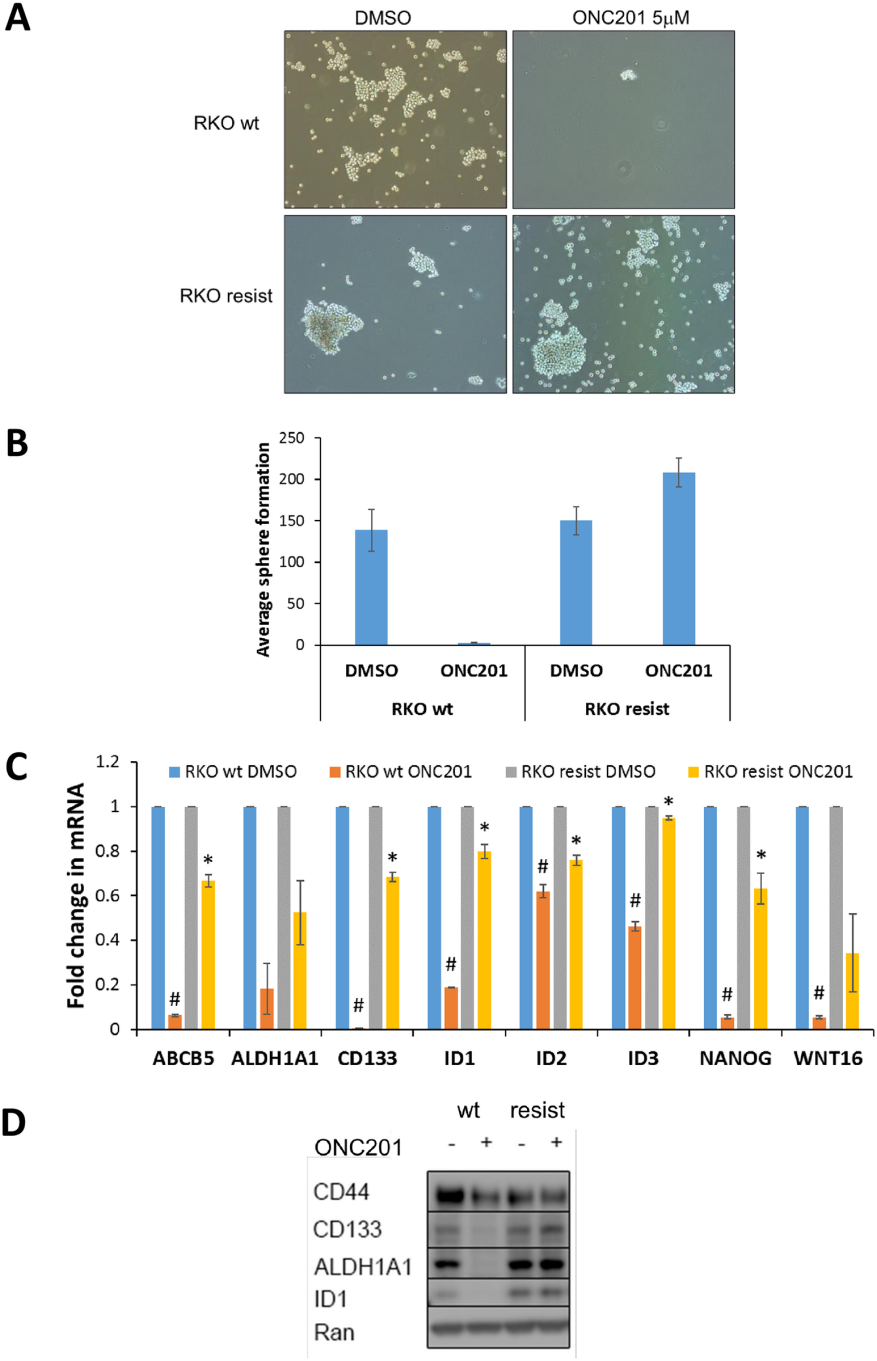
Inhibition of CSCs does not occur in tumor cells with acquired resistance to ONC201. (A) Effect of DMSO/ONC201 (5 μM, 72 h, n = 3) on tumor sphere formation of RKO wild-type (wt) and ONC201-resistant (resist) cells. Representative image (10X magnification) of spheres (> 60 μm) (B) Quantification of spheres in (A). (C) qRT-PCR for indicated stem cell-related genes in DMSO/ONC201-treated (5 μM, 48 h, n = 3) RKO wild-type (wt) and ONC201-resistant (resist) cells. # indicates p < 0.003 relative to wt DMSO. * indicates p < 0.05 relative to wt ONC201. (D) Western blot for indicated stem cell-related proteins in DMSO/ONC201-treated (5 μM, 72 h) RKO wild-type (wt) and ONC201-resistant (resist) cells.

We explored the correlation of ONC201-mediated changes in stem cell-related gene transcription with anti-tumor efficacy. ONC201 inhibited sphere formation of parental RKO wild-type (wt) cells but not RKO cells with acquired resistance to ONC201 (Fig 3A and 3B). Accordingly, ONC201 significantly downregulated mRNA levels of the stem cell-related genes *ID1* (2.1-fold), *FZD4* (1.6-fold), *HES7* (2.5-fold), *CCNB1* (3.7-fold) and *TCF3* (1.8-fold) in RKO wt cells but not in ONC201-resistant RKO cells (S6 Table), indicating that CSC-inhibition could serve as a biomarker of ONC201 response. Validation with qRT-PCR indicated that ONC201-mediated inhibition of CSC-related genes *ABCB5*, *CD133*, *ID1*, *ID2*, *ID3* and *NANOG* in RKO wt cells was significantly reduced in ONC201-resistant RKO cells (Fig 3C). Western blot confirmed that ONC201-mediated downregulation of *CD44*, *CD133*, *ALDH1* and *ID1* occurred in RKO wt cells, but not in ONC201-resistant RKO cells (Fig 3D). Thus, CSC depletion is a critical component of ONC201’s anti-cancer efficacy and can serve as a potential pharmacodynamic biomarker of ONC201 response.

### CSC expression in solid tumors as a potential biomarker of response for ONC201

**Fig 4:**
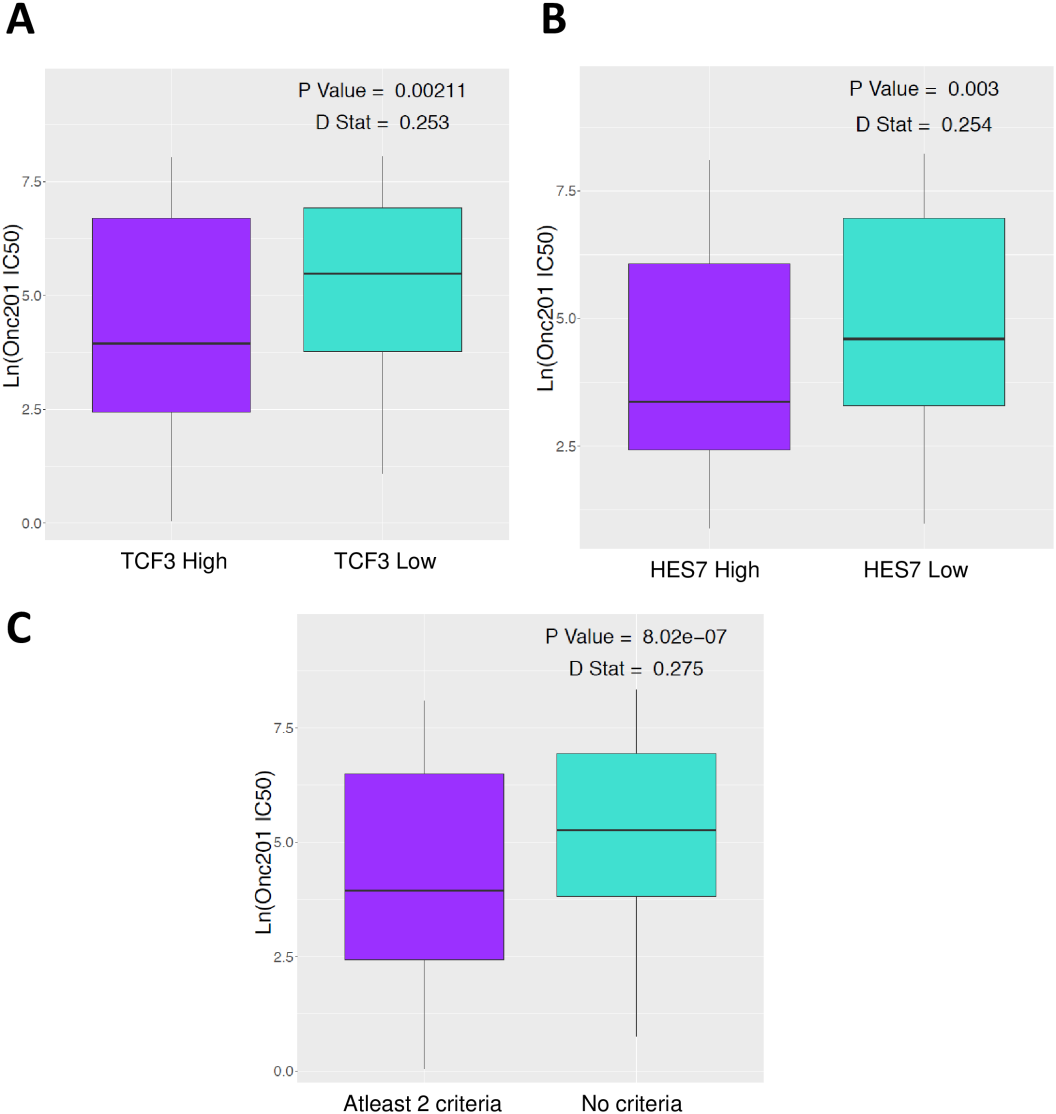
CSC-related gene expression in solid tumors as a potential biomarker of response for ONC201. Distribution of ONC201 efficacy (IC50) in >1000 GDSC cell lines based on basal RNA expression of (A) *TCF3*, (B) *HES7*. (C) Distribution of ONC201 efficacy (IC50) in >1000 GDSC cell lines based on fulfillment of at least two expression based criteria (low expression of *ID1/CD44* and high expression of *TCF3/HES7*) against cell lines that fulfilled none. P value and D statistic are indicated.

Finally, we used the GDSC panel of approximately 1,000 unique cancer cell lines [7] to determine whether ONC201 *in vitro* efficacy correlates with the expression of CSC-related genes in the treatment-naïve setting. All genes identified in the earlier studies were tested and a significant correlation with *ID1* (D stat = 0.18), *CD44* (D stat = 0.173), *TCF3* (D stat = 0.253) and *HES7* (D stat = 0.254) expression was observed (S7 Table). Interestingly we found that high expression of *TCF3* and *HES7* significantly predicted sensitivity to ONC201 (Fig 4A and 4B), suggesting that ONC201 may be efficacious in tumors with high basal Wnt signaling. Also, low expression of *ID1* and *CD44* significantly predicted sensitivity to ONC201 (S1 Fig). These data are consistent with the heterogeneity observed within CSC populations with various combinations of markers representing different cell populations [21]. Furthermore, when we tested ONC201 efficacy in cell lines that fulfilled at least two of the expression based criteria (low expression of *ID1/CD44* and high expression of *TCF3/HES7*) against cell lines that fulfilled none, there was a greater degree of separation (D stat = 0.2749, P-value = 8.02e-07) (Fig 4C). These results indicate that pre-treatment expression of certain CSC genes can serve as predictive biomarkers for ONC201 response and that combining the expression of multiple CSC genes results in a stronger overall prediction.

## Discussion

We have previously demonstrated the anti-CSC efficacy of ONC201 *in vitro* using established CSC markers, sphere cultures and *in vivo* using limiting dilution studies in colorectal cancer [9]. Additionally, ONC201-mediated inhibition of leukemic stem cells has been confirmed *in vivo* [10]. Depletion of chemotherapy-resistant colorectal CSCs by ONC201 involves an Akt-ERK-Foxo3-DR5-TRAIL-dependent mechanism of inhibition of self-renewal and cell death induction [9, 11]. However, it was unclear whether ONC201 depletion of CSCs is a consequence of cell death or involves specific effects on stem-cell related genes that precede inhibition of self-renewal and cell death. In this study, we show that ONC201 specifically impacts stem cell-related transcription at time points (18 and 48 h) that precede cell death which occurs 60-72h post treatment in solid tumor cells [11]. These early effects on stem-cell related transcription are followed by inhibition of CSC markers and self-renewal by ONC201 at 60–72 h [9].

ONC201 attenuates diverse CSC markers such as *CD44*, *CD133*, *ABCB5*, *ALDH1A1*, *ALDH7A1*, *NANOG*, *ID1*, *ID2*, *ID3* [2, 3] and self-renewal signaling pathways such as Wnt, Notch and Hedgehog [15, 16, 19] that drive tumor-initiation [13, 21], therapy resistance [17] and metastasis [14, 18] across various tumor types providing an opportunity for broad-spectrum anti-CSC and anti-cancer effects. The ability to target established mechanisms of CSC chemotherapy resistance such as ABCB5 is consistent with previously demonstrated ONC201 efficacy in 5-Fluorouracil resistant colorectal CSC models [9]. Gene expression profiles in colorectal cancer cells revealed ONC201 targets CSC genes involved in prostate cancer and glioblastoma. Accordingly, ONC201 mediated inhibition of self-renewal in solid tumors was confirmed in prostate cancer cell lines and glioblastoma patient derived cells. This study provides further evidence of the broad spectrum anti-cancer efficacy of ONC201 and serves as a rationale for the ongoing single agent Phase I/II trials of ONC201 in advanced refractory solid tumors including prostate cancer and glioblastoma [7]. Drugs targeting differentiated bulk tumor cells alone are typically associated with early clinical responses that may or may not be durable. In contrast, CSC-targeting agents are likely to achieve delayed but durable responses [22]. ONC201’s ability to target CSCs provides an opportunity to potentially achieve durable responses in patients with advanced therapy resistant disease, especially in high unmet need indications such as recurrent glioblastoma. Additionally, approved chemotherapies or targeted agents with anti-proliferative effects that do not target CSCs could be combined with ONC201 to provide rapid de-bulking and durable clinical benefit. These results also indicate that ONC201 could be used in the adjuvant/preventative setting for cancer recurrence and metastasis prevention.

To allow clinical translation of our results, we show that CSC-related gene expression can serve as a potential biomarker of ONC201 response. To demonstrate utility as a pharmacodynamic biomarker, cells with acquired resistance to ONC201 previously generated in our lab were used [12]. ONC201 mediated CSC inhibition occurs in sensitive but not in cancer cells with acquired resistance, as confirmed by sphere formation, gene expression and protein levels of established CSC markers. To demonstrate utility as a predictive biomarker, we used the GDSC panel of cancer cell lines [7]. Interestingly, baseline expression of CSC-related genes including high basal Wnt signaling predicted ONC201 anti-cancer efficacy in >1000 cancer cell lines. Thus, correlative studies testing CSC expression at the RNA and protein level using circulating tumor cells and biopsies from ongoing ONC201 clinical studies are warranted.

## Supporting information

S1 Table List of qRT-PCR primers. (XLSX)

S2 Table Stem cell transcription factor changes identified with Ingenuity pathway analysis for gene expression profiles of HCT116 p53-null cells treated with ONC201 (10 μM) for 48h. Fold change relative to DMSO treated cells.
(XLS)

S3 Table Changes in stem cell-related genes identified with Ingenuity pathway analysis for gene expression profiles of HCT116 p53-null cells treated with ONC201 (10 μM) for 48 h. Fold change relative to DMSO treated cells.
(XLS)

S4 Table Ingenuity pathway analysis for gene expression profiles of HCT116 cells treated with ONC201 (5 μM) for 18 h and 48 h. (XLS)

S5 Table Ingenuity pathway analysis for gene expression profiles of RKO cells treated with ONC201 (5 μM) for 48 h. (XLS)

S6 Table Stem cell-related changes in gene expression in RKO wild-type and ONC201-resistant cells treated with ONC201 (5 μM) for 48 h. Fold change relative to DMSO treated cells.
(XLSX)

S7 Table P value and D statistic for correlation of ONC201 efficacy in GDSC screen with pretreatment expression of select CSC-related genes. (XLSX)

S1 Fig ONC201 targets CSCs in prostate and glioblastoma tumors. qRT-PCR for indicated stem cell-related genes in DMSO/ONC201-treated (5 μM, 24h/48h, n = 3) (A) T98G and (B) U251 cells. * indicates p < 0.02 relative to DMSO. (C) and (D) Western blot for indicated stem cell-related proteins in glioblastoma cells treated with indicated doses of DMSO/ONC201 for indicated time. (E) Western blot for indicated proteins in DMSO/5 μM ONC201-treated 22Rv1 cells for indicated time. (F) Western blot for indicated proteins in DMSO/ONC201-treated LNCaP cells for 72 h. (G) Distribution of ONC201 efficacies in GDSC cancer cells based on basal RNA expression of *ID1* and (H) *CD44*.
(TIF)
