# Supplementary figures and images for: Cancer stem cell-related gene expression as a potential biomarker of response for first-in-class imipridone ONC201 in solid tumors
Source: PLoS One. 2017 Aug 2;12(8):e0180541. doi: 10.1371/journal.pone.0180541 (PMC5540272; doi:10.1371/journal.pone.0180541)

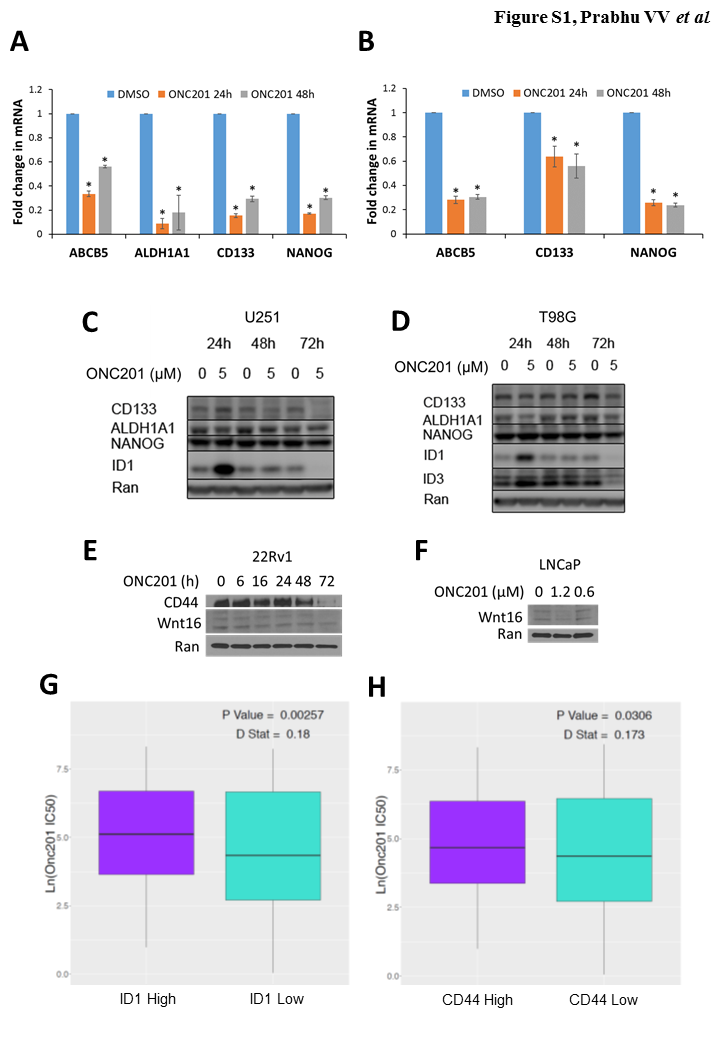

Supplement: S1 Fig — qRT-PCR for indicated stem cell-related genes in DMSO/ONC201-treated (5 μM, 24h/48h, n = 3) (A) T98G and (B) U251 cells. * indicates p < 0.02 relative to DMSO. (C) and (D) Western blot for indicated stem cell-related proteins in glioblastoma cells treated with indicated doses of DMSO/ONC201 for indicated time. (E) Western blot for indicated proteins in DMSO/5 μM ONC201-treated 22Rv1 cells for indicated time. (F) Western blot for indicated proteins in DMSO/ONC201-treated LNCaP cells for 72 h. (G) Distribution of ONC201 efficacies in GDSC cancer cells based on basal RNA expression of ID1 and (H) CD44. (TIF) [file pone.0180541.s008.tif]
